# Mucosal Snare Resection (MSR) in Non-Submucosal Injection Endoscopic Submucosal Excavation (NSI-ESE) for submucosal tumor may not be the preferable choice: A retrospective study

**DOI:** 10.1371/journal.pone.0343335

**Published:** 2026-02-20

**Authors:** Jiatao Tu, Lixing Yu, Kaihan Wu, Meng Gu, Chencong Zhou, Xuan Huang

**Affiliations:** Department of Gastroenterology, The First Affiliated Hospital of Zhejiang Chinese Medical University, No. 54, Youdian Road, Shangcheng District, Hangzhou, Zhejiang, China; Dalin Tzu Chi Hospital, Buddhist Tzu Chi Medical Foundation, TAIWAN

## Abstract

**Background and aims:**

Non-submucosal injection endoscopic submucosal excavation (NSI-ESE) and mucosal snare resection-assisted endoscopic submucosal excavation (MSR-ESE) are novel techniques for the treatment of gastric submucosal tumors (SMTs). This study aims to evaluate the feasibility, safety, and efficacy differences between these two methods in the management of gastric SMTs.

**Methods:**

A retrospective analysis was conducted on 95 eligible patients who underwent endoscopic treatment for gastric SMTs between 01/01/2022 and 31/12/2024, including 41 patients treated with MSR-ESE and 54 with NSI-ESE. Differences in operative time, safety, and cost-effectiveness between the two groups were compared, and multivariate linear regression analysis was performed to investigate the independent impact of the surgical approach on operative time.

**Results:**

There were no significant differences between the two groups in baseline characteristics, en bloc resection rate, or incidence of adverse events such as intraoperative perforation and delayed bleeding. However, the MSR-ESE group had significantly longer tumor exposure time (6.37 ± 2.98 min vs. 4.61 ± 2.94 min, p = 0.001), tumor excavation time (29.59 ± 9.09 min vs. 24.09 ± 9.87 min, p = 0.007), and total procedure time (35.95 ± 10.23 min vs. 30.63 ± 11.61 min, p = 0.022) compared with the NSI-ESE group. Multivariate regression analysis confirmed that MSR-ESE was an independent factor associated with prolonged tumor exposure time (β = 1.60, p = 0.005) and total procedure time (β = 5.43, p = 0.012). In addition, the surgery-related cost was significantly higher in the MSR-ESE group than in the NSI-ESE group (US$874.94 ± 106.40 vs. US$731.90 ± 108.98, p < 0.038).

**Conclusion:**

In the treatment of gastric SMTs, MSR-ESE did not demonstrate any advantages in efficiency or safety compared with NSI-ESE; instead, it resulted in a significantly longer procedure time, more discomfort and increased economic burden. Therefore, the routine use of snare-assisted mucosal resection during ESE is not recommended in clinical practice.

## 1. Introduction

Gastric submucosal tumors (SMTs) are defined as masses or protrusions covered by normal gastric mucosa. Most SMTs are small in size and asymptomatic, often detected incidentally during endoscopic or radiological examinations [1]. Although the majority of SMTs are considered benign, the potential for malignant transformation warrants clinical vigilance [2,3]. Due to the intact mucosal covering, conventional biopsy techniques often fail to yield diagnostic tissue samples. Furthermore, the accuracy of endoscopic diagnosis is highly operator-dependent, and prolonged surveillance may contribute to increased psychological stress for patients. Accordingly, several clinical guidelines advocate for early interventional management in selected cases to mitigate the risk of malignancy [4].

Endoscopic submucosal excavation (ESE) has emerged as a common therapeutic approach for SMTs, owing to its minimally invasive nature and favorable safety profile [5]. This procedure typically involves submucosal injection of a methylene blue–glycerol fructose mixture to create an operative space [6]. However, such injections may inadvertently lead to tumor displacement, thereby increasing the technical complexity of resection. To overcome these limitations, non-submucosal injection ESE (NSI-ESE) has been developed [7]. In this technique, an ESD knife is used to incise the surrounding mucosa, followed by direct dissection along the muscularis propria. This modification has been shown to significantly reduce operative time and enhance procedural safety [7].

Recently, an innovative technique known as mucosal snare resection-assisted ESE (MSR-ESE) has been introduced [8]. Based on NSI-ESE, this method employs a snare to resect the overlying mucosa without the need for marking or submucosal injection. Preliminary studies suggest that MSR-ESE offers a substantial reduction in operative time compared to conventional ESE [9].

Despite these advances, there is a paucity of systematic comparative studies evaluating NSI-ESE and MSR-ESE. Therefore, the present study aims to assess the clinical feasibility and safety profiles of these two modified techniques through a retrospective cohort analysis.

## 2. Methods

### 2.1. Study population

This retrospective study included patients with gastric submucosal tumors who underwent endoscopic submucosal excavation (ESE) at the Endoscopy Center of Zhejiang Hospital of Traditional Chinese Medicine between 01/01/2022 and 31/12/2024. Patients with non-neoplastic lesions (n = 6), those who underwent conventional submucosal injection ESE (n = 42), and those with incomplete clinical data (n = 11) were excluded. All patients underwent preoperative evaluation with computed tomography (CT) and endoscopic ultrasonography (EUS) to assess tumor size and originating layer. Treatment decisions were made following discussions among senior endoscopists. All ESE procedures were performed by experienced endoscopists with over 5 years of expertise in endoscopic submucosal dissection (ESD) and an annual volume of over 25 ESD cases. This research has been approved by the Ethics Review Committee of Zhejiang Hospital of Traditional Chinese Medicine (2025-KL-068–01). Written informed consent was obtained from all participants prior to inclusion in the study. All data were obtained on 01/04/2025.

### 2.2. Surgical procedure

All patients fasted for at least 12 hours prior to the procedure. Under standard left lateral decubitus positioning, anesthesia was induced with intravenous propofol followed by endotracheal intubation. Vital signs, including heart rate, blood pressure, oxygen saturation, and airway pressure, were continuously monitored throughout the procedure.

**Fig 1:**
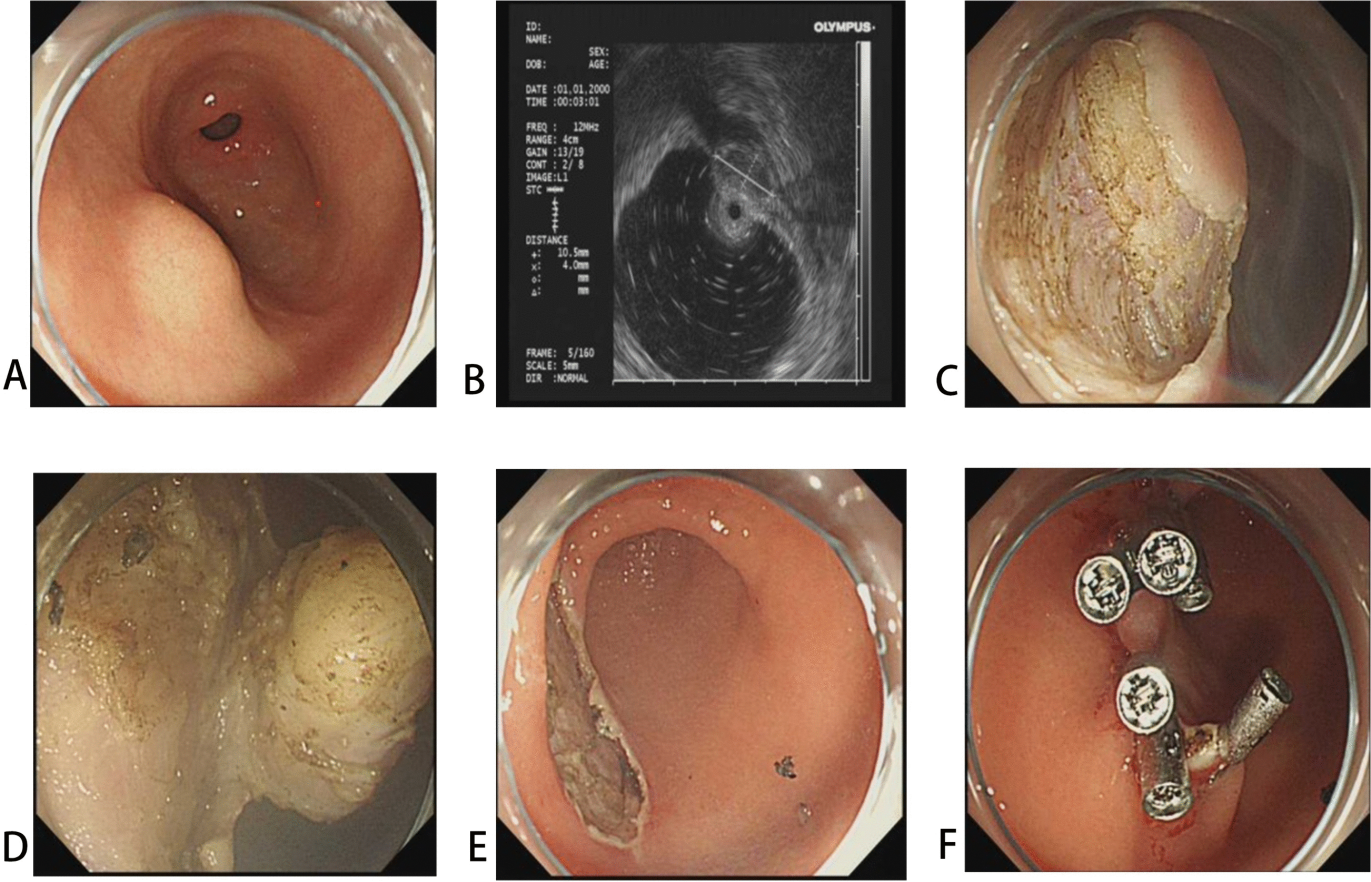
The non-submucosal injection endoscopic submucosal excavation (NSI-ESE) of a gastric SMT. **(A)** An SMT was observed in corpus. **(B)** EUS shows that the lesion originates from the muscularis propria layer. **(C)** Without submucosal injection, the mucosa is directly incised circumferentially around the lesion. **(D-E)** Dissection is performed along the base of the lesion and En bloc resection was achieved. **(F)** The mucosal defect was closed with hemostatic clips.

The procedural steps for NSI-ESE were as follows (Fig 1): After assessment of the lesion’s size and layer of origin using EUS (GF-EU260, Olympus Co., Ltd.), the mucosal layer was incised to fully expose the tumor margins and base using a dual knife (KD-650L, Olympus Co., Ltd.). Dissection was carried out along the anatomical plane between the tumor base and the muscularis propria by a dual knife or an insulation-tipped knife (KD-612L, Olympus Co., Ltd.). For deeply invasive or extraluminally growing lesions, especially those adherent to the serosa, endoscopic full-thickness resection (EFTR) was performed under traction to ensure en bloc resection. Hemostasis was achieved by coagulating exposed vessels, and the wound and perforation sites were closed using titanium clips (ROCC-D-26–195-C, Micro-Tech (Nanjing) Co., Ltd.). For cases with perforation, a nasogastric tube was placed for gastrointestinal decompression.

**Fig 2:**
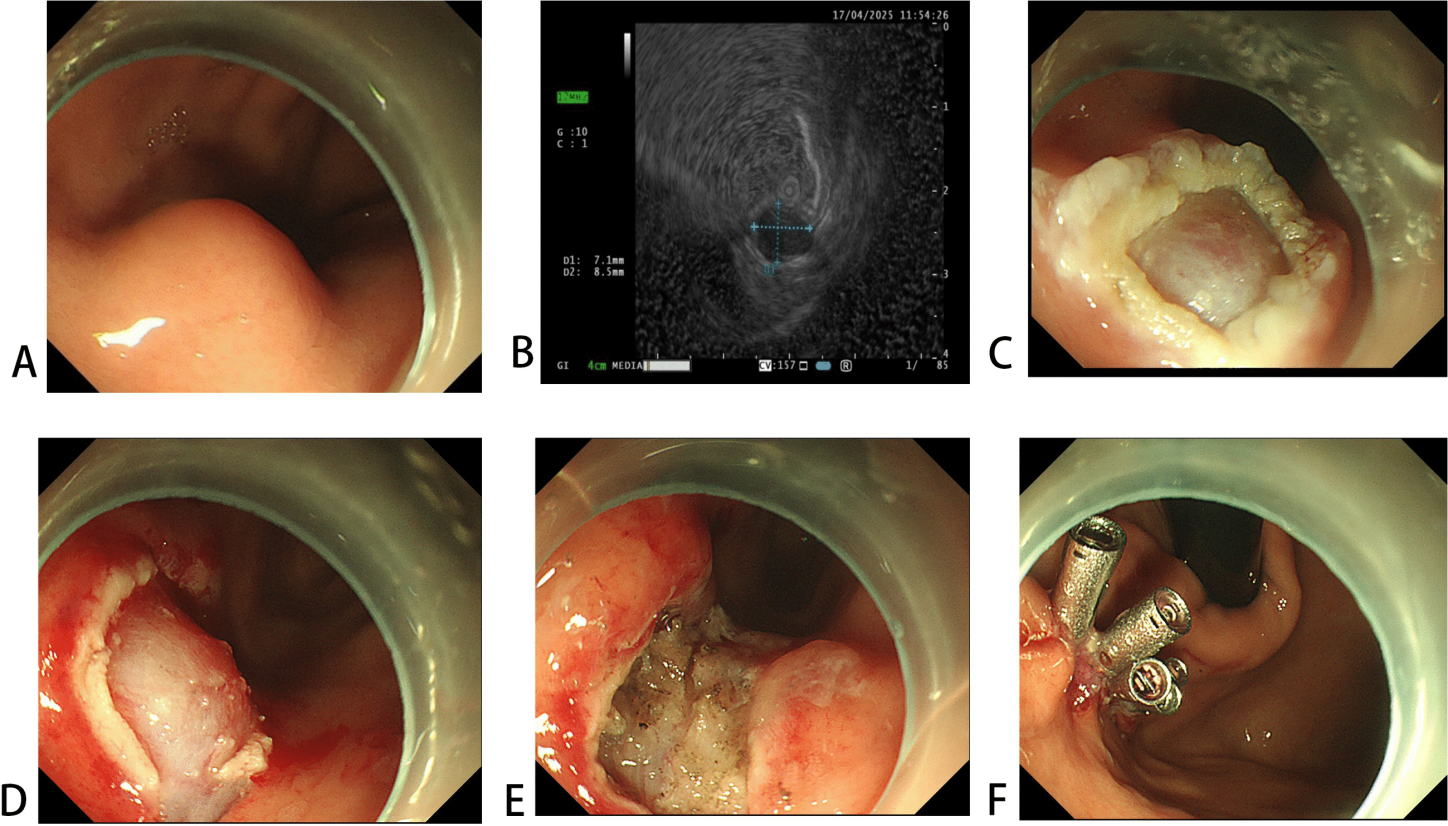
The procedure of mucosal snare resection endoscopic submucosal excavation (MSR-ESE). **(A)** An SMT is observed. **(B)** The tumor originates from the muscularis propria layer on EUS. **(C)** Without submucosal lifting, The superficial mucosa over the lesion was partly resected with a snare. **(D)** The margins were extended and the tumor fully exposed using a Dual Knife. **(E-F)** En bloc resection of the lesion was achieved, and the wound was closed with hemostatic clips.

The key difference between NSI-ESE and MSR-ESE lies in the initial mucosal handling step. The procedural steps for MSR-ESE were as follows (Fig 2): After EUS evaluation, without submucosal injection, the mucosa overlying the tumor was resected by electrocautery using a snare (SD-230U, Olympus Co., Ltd.). Residual mucosa was removed with a dual knife or an insulation-tipped knife to fully expose the tumor, which was then dissected en bloc along the anatomical plane between the tumor and muscularis propria.

### 2.3. Postoperative management

After the procedure, patients were kept strictly nil per os (NPO) and gradually transitioned to a semi-liquid diet based on the recovery of gastrointestinal function. Routine monitoring included vital signs such as body temperature, blood pressure, heart rate, and oxygen saturation. Supportive treatment consisted of intravenous fluid replacement, proton pump inhibitor (PPI) infusion, and oral administration of gastric mucosal protective agents. Laboratory tests—including complete blood count, C-reactive protein, liver and renal function, and coagulation profile—were regularly reviewed. Patients were closely observed for warning signs such as significant abdominal pain or hematochezia, which could indicate serious complications like delayed perforation or gastrointestinal bleeding. Prophylactic antibiotics were not routinely administered unless there was clear evidence of infection.

### 2.4. Histopathological examination

All resected specimens were fixed in 10% formalin, embedded in paraffin, and submitted to the pathology department for histopathological evaluation. En bloc resection was defined as removal of the tumor without rupture. Histopathological classifications primarily included GISTs, leiomyomas, lipomas, fibromas, schwannomas, Brunner’s gland hamartomas, and ectopic pancreas [10].

### 2.5. Data collection

Baseline data were extracted from the hospital’s electronic medical record system, including patient age, sex, surgical approach, EFTR rate, tumor size, location, originating layer, pathological diagnosis, and laboratory parameters. Surgery-related indicators, including tumor exposure time, dissection time, total procedure time, and en bloc resection rate, were used to evaluate clinical efficacy. The highest postoperative numerical rating scale (NRS) pain score within 24 hours was used to evaluate patients’ subjective perception. Postoperative fasting duration and length of hospital stay were used to evaluate postoperative recovery. The rate of intraoperative perforation and postoperative adverse events was used to assess surgical safety. Surgery-related medical costs and total treatment-related costs were used to evaluate economic outcomes.

Time of tumor exposure was defined as the time required to fully expose the tumor margins and base. Time of excavation referred to the duration needed to completely separate the tumor from the gastric wall. Surgical time was defined as the total time from the beginning of the operation to the completion of wound management and withdrawal of the endoscope. En bloc resection was defined as complete removal of the lesion in a single piece during endoscopy. EFTR was performed when full-thickness resection of the gastric wall was required due to either intentional or accidental perforation. Adverse events included intraoperative perforation, delayed perforation and delayed bleeding. Gastrointestinal function recovery time and hospital stay were defined as the time to resume a semi-liquid diet and time to discharge, respectively. Surgery-related costs included medication, surgical instruments, anesthesia, and nursing care during the procedure. Total treatment-related cost was defined as all expenses incurred during the patient’s hospital stay for undergoing ESE.

### 2.6. Statistical analysis

Continuous variables were expressed as mean ± standard deviation. If the data followed a normal distribution, comparisons between groups were performed using the *t*-test; otherwise, the Mann–Whitney *U* test was used for non-normally distributed data. Categorical variables were presented as counts and percentages [n (%)], and comparisons between groups were conducted using the chi-square test or Fisher’s exact test, as appropriate.

Two regression models were constructed to further investigate the impact of surgical approach on surgical time: Model 1 was a univariate regression analysis, and Model 2 was a multivariate regression analysis adjusting for histological type, tumor location, morphology, and tumor size.

A *p*-value < 0.05 was considered statistically significant. All statistical analyses were conducted using SPSS Statistics version 29.0.2.0.

## 3. Results

### 3.1. Patient characteristics

Between 01/01/2022 and 31/12/2024, a total of 154 patients underwent ESE for gastric SMTs. After excluding 59 patients, a total of 95 patients were included in this study. Among them, 41 underwent MSR-ESE and 54 received NSI-ESE. In the MSR-ESE group, there were 15 males (36.6%) and 26 females (63.4%), with a mean age of 56.02 ± 11.79 years. In the NSI-ESE group, 14 patients were male (25.9%) and 40 were female (74.1%), with a mean age of 54.98 ± 11.98 years. All patients underwent white-light endoscopy and EUS prior to treatment. Tumor locations included the gastric fundus (50 cases, 52.6%), gastric body (26 cases, 27.4%), cardia (12 cases, 12.6%), and antrum (7 cases, 7.4%). Tumor morphology was classified as sessile (66 cases, 69.47%), flat (19 cases, 20.00%), or pedunculated (10 cases, 10.53%), with no significant difference between groups. Postoperative pathological diagnoses revealed 47 cases of leiomyoma (49.5%), 37 cases of gastrointestinal stromal tumor (GIST) (38.9%), 9 cases of lipoma (9.5%), and 1 case each of schwannoma and fibroma (1.1%). There were no statistically significant differences in baseline characteristics between the two groups, including age, sex, tumor location, maximum diameter, pathological type, BMI, comorbidities (hypertension), or coagulation parameters (platelet count and INR) (all P > 0.05). A difference was observed in diabetes mellitus between the two groups (P < 0.05). Detailed information is presented in Table 1.

**Table 1:** Baseline table.

Characteristic	NSI-ESE N = 54	MSR-ESE N = 41	p-value
Age (Years)	54.98 ± 11.98	56.02 ± 11.79	0.673
Gender, n (%)			0.264
Male	14 (25.9%)	15 (36.6%)	
Female	40 (74.1%)	26 (63.4%)	
Hypertension	23 (43%)	12 (29%)	0.182
Diabetes	9 (17%)	3 (7.2%)	0.025
BMI (kg/m²)	28.09 ± 5.19	27.83 ± 5.51	0.814
PLT (10^9/L)	230.35 ± 53.71	234.07 ± 45.75	0.854
INR	1.14 ± 0.24	1.11 ± 0.23	0.528
Clinicopathological types, n (%)			0.560
Stromal tumors	18 (33%)	19 (46%)	
Leiomyomas	29 (54%)	18 (44%)	
Lipoma	5 (9.3%)	4 (9.8%)	
Schwannoma	1 (1.9%)	0 (0%)	
Fibromyxoma	1 (1.9%)	0 (0%)	
Tumor location, n (%)			0.967
Cardia	6 (11%)	6 (15%)	
Fundus	29 (54%)	21 (51%)	
Corpus	15 (28%)	11 (27%)	
Antrum	4 (7.4%)	3 (7.3%)	
Morphology, n (%)			0.773
Sessile	37 (68.5%)	29 (70.7%)	
Flat	12 (22.2%)	7 (17.1%)	
Pedunculated	5 (9.3%)	5 (12.2%)	
Long axis (cm)	1.15 ± 0.48	1.10 ± 0.46	0.650
Short axis (cm)	0.84 ± 0.39	0.88 ± 0.44	0.694
Tumor Size, n (%)			0.623
> 10mm	21 (39%)	18 (44%)	
≤ 10 mm	33 (61%)	23 (56%)	

### 3.2. Outcomes

A comparative analysis of surgical outcomes between the NSI-ESE and MSR-ESE groups (Table 2) revealed no significant differences in the rates of EFTR [33.33% vs. 26.47%, p = 0.495] and intentional perforation [27.78% vs. 24.39%, p = 0.710]. The mean time to tumor exposure was significantly shorter in the NSI-ESE group compared to the MSR-ESE group (4.61 ± 2.94 min vs. 6.37 ± 2.98 min, p = 0.001), indicating that snare-assisted mucosal resection did not improve the efficiency of lesion exposure. Similarly, the time of excavation (24.09 ± 9.87 min vs. 29.59 ± 9.09 min, p = 0.007) and total surgical time (30.63 ± 11.61 min vs. 35.95 ± 10.23 min, p = 0.022) were both significantly shorter in the NSI-ESE group.

**Table 2:** Surgical Outcome.

Outcome	NSI-ESE	MSR-ESE	p-value
Performed EFTR, n (%)	18 (33.33%)	11 (26.47%)	0.495
Intentional perforation, n (%)	15 (27.78%)	10 (24.39%)	0.710
Time of tumor exposure (mins)	4.61 ± 2.94	6.37 ± 2.98	0.001
Time of excavation (mins)	24.09 ± 9.87	29.59 ± 9.09	0.007
Surgical time (mins)	30.63 ± 11.61	35.95 ± 10.23	0.022
Highest pain score	0.80 ± 0.81	1.29 ± 1.17	0.042
Gastrointestinal function recovery time (days)	2.61 ± 0.76	2.37 ± 1.02	0.205
Postoperative hospital stay (days)	4.52 ± 2.01	4.20 ± 1.44	0.547
Surgery-related costs ($)	731.90 ± 108.98	874.94 ± 106.40	0.038
Total treatment-related costs ($)	1535.81 ± 183.41	1643.03 ± 59.18	< 0.001

Pain scores were used to evaluate the patients’ subjective perception of pain. Patients in the MSR-ESE group had significantly higher pain scores than those in the NSI-ESE group (0.80 ± 0.81 vs. 1.29 ± 1.17, P = 0.042). Postoperative recovery indicators, including gastrointestinal function recovery time (2.61 ± 0.76 days vs. 2.37 ± 1.02 days, p = 0.205) and hospital stay (4.52 ± 2.01 days vs. 4.20 ± 1.44 days, p = 0.547), were comparable between groups. However, surgery-related costs were significantly higher in the MSR-ESE group compared to the NSI-ESE group ($874.94 ± 106.40 vs. $731.90 ± 108.98, p = 0.038) In addition, the total treatment-related costs were also higher in the MSR-ESE group ($1643.03 ± 59.18 vs. $1535.81 ± 183.41, p < 0.001).

### 3.3. Linear regression analysis

To account for other factors and assess the independent effect of the surgical approach on surgery-related time, we conducted linear regression analysis. As shown in Table 3, linear regression analysis revealed that MSR-ESE was significantly associated with a longer time of tumor exposure compared to NSI-ESE in both univariate (β = 1.75, 95% CI: 0.54–2.97, p = 0.005) and multivariate models (β = 1.60, 95% CI: 0.51–2.68, p = 0.005). Similarly, the time of excavation was significantly longer in the MSR-ESE group in both univariate (β = 5.49, 95% CI: 1.57–9.42, p = 0.007) and multivariate analyses (β = 5.64, 95% CI: 2.09–9.21, p = 0.003). Moreover, the total surgical time was also significantly prolonged in the MSR-ESE group in both univariate (β = 5.32, 95% CI: 0.78–9.86, p = 0.022) and multivariate models (β = 5.43, 95% CI: 1.29–9.58, p = 0.012).

**Table 3:** Liner regression.

Surgical-related Time	Univariate regression			Multivariate regression		
	β^1^	95% (CI)^1^	p-value	β^1^	95% (CI)^1^	p-value
Time of tumor exposure	1.75	0.54, 2.97	0.005	1.60	0.51, 2.68	0.005
Time of excavation	5.49	1.57, 9.42	0.007	5.64	2.09, 9.21	0.003
Surgical time	5.32	0.78, 9.86	0.022	5.43	1.29, 9.58	0.012

^1^NSI-ESE as the Reference

### 3.4. Adverse events

The incidence of adverse events was low in both groups (Table 4). Accidental perforation occurred in 3 patients (5.56%) in the NSI-ESE group and in 1 patient (2.44%) in the MSR-ESE group, with no statistically significant difference (*p* = 0.631). No cases of delayed perforation were observed in either group. Delayed bleeding occurred in one case (2.44%) in the MSR-ESE group and was absent in the NSI-ESE group (*p* = 0.989).

**Table 4:** Adverse events.

Adverse events	NSI-ESE	MSR-ESE	p-value^3^
Accidental perforation	3 (5.56%)	1 (2.44%)	0.631
Delayed perforation	0 (0%)	0 (0%)	
Delayed bleeding	0 (0%)	1 (2.44%)	0.989

## 4. Discussion

Open or laparoscopic surgery was once the standard treatment for gastric submucosal tumors (SMTs), particularly GISTs [11]. However, unlike gastric cancer, SMTs typically do not require extended resection or lymph node dissection unless there are clear signs of malignancy. Therefore, the primary treatment goal is complete tumor removal to eliminate the risk of malignant transformation [12]. With advances in endoscopic techniques, endoscopic surgery has emerged as a less invasive and cost-effective alternative for the resection of small SMTs. Current approaches include ESE, EFTR, and submucosal tunneling endoscopic resection (STER) [13]. Among them, STER relies on the creation of a submucosal tunnel, making it more suitable for lesions in tubular structures such as the esophagus or rectum. MSR-ESE is a snare-assisted modification of NSI-ESE. In this study, we compared the efficacy, safety, and cost-effectiveness of these two endoscopic approaches in the treatment of gastric SMTs. Our results indicated that the two techniques achieved similar safety and therapeutic outcomes; nonetheless, MSR-ESE resulted in increased surgical expenses and reduced procedural efficiency.

**Fig 3:**
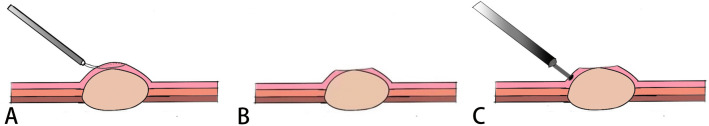
Limitations of snare-assisted ESE. **(A)** The snare fails to grasp the tumor base when the lesion is large or non-protruding. **(B)** Only partial mucosal removal is achieved. **(C)** An ESD knife is needed to fully expose the tumor margins.

Snares can be used for the resection of certain small SMTs [14]. However, in clinical practice, when the tumor is relatively large or does not markedly protrude into the gastric lumen, it can be challenging for the snare to securely grasp the tumor base during tightening (as shown in Fig 3A). In such cases, only a portion of the superficial mucosa can be removed (Fig 3B), making the use of the snare alone ineffective. Although the exposed mucosal defect allows the operator to visually locate the lesion, an ESD knife is still required to further enlarge the incision until the tumor margins are fully exposed (Fig 3C). In contrast, with preoperative EUS used to evaluate tumor size and depth, making an incision directly along the premarked points can improve the efficiency of this step. Furthermore, we observed that repeated snare-assisted mucosal resection led to local mucosal carbonization and edema, resulting in a blurred operative field and greater technical difficulty during tumor dissection, which could contribute to the lower efficiency of MSR-ESE. Moreover, in our study, although neither group experienced pain scores greater than 5 within 24 hours after the procedure, patients in the MSR-ESE group reported higher pain scores. Previous studies have indicated that postoperative pain may be associated with longer procedure duration [15].

The rate of en bloc resection is also an important indicator of surgical efficacy. Besides tumor size and mitotic index, tumor rupture is a known risk factor for GIST recurrence [16]. In this study, all SMTs were resected en bloc under endoscopy, with no gross tumor rupture observed. We did not assess the R0 resection rate, as Moon Kyung Joo et al. reported an R0 resection rate of only 25.6% in endoscopic treatment of small GISTs, yet with a very low recurrence rate during long-term follow-up [17]. Another study also demonstrated that tumor characteristics, tumor size, and gross en bloc resection have a greater impact on prognosis, whereas a microscopically positive resection margin (R1) does not appear to adversely affect outcomes [18].

In terms of safety, the use of snares in the MSR-ESE group did not lead to a significantly higher incidence of adverse events compared with the NSI-ESE group. ESE, unlike endoscopic submucosal dissection (ESD), enables resection of lesions originating from the deeper muscularis propria, which inherently carries a higher risk of intraoperative perforation—reported to be approximately 33% in the literature [19]. In our study, the overall perforation rate was 30.74% (29/95), which is comparable to recent reports of ESE performed with submucosal injection [20]. Therefore, the absence of submucosal injection did not appear to increase procedural risk. Submucosal injection creates a fluid cushion that is essential for resecting lesions confined to the mucosal layer, as it separates the mucosa from the muscularis propria and reduces the risks of perforation and thermal injury [21]. However, in the context of SMT resection, submucosal injection cannot effectively separate the tumor from the muscularis propria and may even cause tumor displacement. Repeated injections may prolong surgical time and potentially lead to tissue injury (e.g., from hypertonic glucose). Thus, injection is unnecessary during ESE for SMTs.

Tumors arising from deep layers, those exhibiting extraluminal growth, or those with tight adhesion to surrounding tissues pose challenges to endoscopic resection and often require EFTR for complete removal. Existing studies support the safety and efficacy of EFTR [22,23], and the use of clips or nylon sutures for post-EFTR closure does not significantly increase the overall cost [7]. Therefore, planned perforation during EFTR is not considered an adverse event [24].

We also observed a significant difference in surgery-related costs and total treatment-related costs between the two groups. The Diagnosis-Related Groups Prospective Payment System (DRG-PPS), which is widely adopted in many developed countries, effectively reduces inpatient expenses and the burden on healthcare systems [25]. It is also a key focus of China’s current healthcare reform, encouraging clinicians to adopt the most cost-effective treatment strategies. Since the snare in MSR-ESE serves only as an auxiliary tool and the tumor resection still requires at least one ESD knife, MSR-ESE results in higher material costs compared to NSI-ESE. Beyond the direct treatment-related costs, hospitalization may lead to indirect economic burdens resulting from inability to work; nevertheless, no significant difference in hospital stay was observed between the two groups.

This is the first study to comprehensively compare MSR-ESE and NSI-ESE for the treatment of gastric SMTs from multiple perspectives. However, several limitations should be acknowledged. First, the retrospective and single-center design, together with the relatively small sample size, may limit the generalizability of the findings to broader clinical settings. Second, the choice of operative technique was determined by the endoscopists rather than by random allocation, which introduces potential operator-dependent selection bias. In addition, the absence of microscopic margin assessment (R0/R1) limits our ability to fully evaluate the oncologic completeness of the procedures. Finally, although all procedures were performed by experienced endoscopists using an established technique, subtle operator learning curve effects on procedural time cannot be completely excluded.

Therefore, future studies should aim to address these limitations by conducting prospective, multicenter, randomized trials with larger sample sizes, standardized operative protocols, and systematic pathological margin assessment. Such studies would provide more robust evidence regarding the comparative safety, efficacy, and long-term oncologic outcomes of MSR-ESE and NSI-ESE.

## 5. Conclusion

In conclusion, compared with NSI-ESE, MSR-ESE not only failed to demonstrate higher safety but also resulted in lower efficiency, more discomfort and greater economic burden. Therefore, based on our findings, routine use of a snare to remove the overlying mucosa prior to NSI-ESE is not recommended.

## Supporting information

S1 File Supplementary materials. This file contains the research protocol and the data used in this study.
(ZIP)
