# Supplementary material for: Mucosal Snare Resection (MSR) in Non-Submucosal Injection Endoscopic Submucosal Excavation (NSI-ESE) for submucosal tumor may not be the preferable choice: A retrospective study
Source: PLoS One. 2026 Feb 20;21(2):e0343335. doi: 10.1371/journal.pone.0343335 (PMC12923029; doi:10.1371/journal.pone.0343335)
Supplement: S1 File — This file contains the research protocol and the data used in this study. (ZIP) [file pone.0343335.s001.zip › Research protocol.docx]

Research protocol

Version number: vOGA20241209

Version Date: 2024-12-09

1. **Project Name**: Efficacy and safety analysis of mucosal snarter resection combined with endoscopic submucosal excision in the treatment of gastric submucosal tumors
2. **Background**

Endoscopic submucosal excision (ESE) is a minimally invasive surgical excision treatment to remove gastric submucosal swelling. The routine surgical steps of this technique all involve cauterization of the marker to delineate the target lesion, followed by submucosal injection and removal of the lesion using a hook knife, insulating knife, or double knife. Endoscopic mucosal snare resection combined with submucosal excision (MSR-ESE) is the latest reported treatment, but dozens of cases have been carried out in the endoscopy center of our hospital, which has the advantages of lower technical burden and shorter operation time, and its steps include (1) surrounding the superficial mucosa of the lesion with a snare and performing electrocoagulation resection without prior submucosal injection; (2) Use an insulating knife to peel off the lesion (3) Use a metal clip to close the wound. However, there is currently a lack of systematic analysis of its effectiveness and safety.

1. **Objectives of the study**

This study intends to compare the efficacy and safety of mucosal snare resection combined with endoscopic submucosal excision and conventional submucosal excision in the treatment of gastric submucosal tumors through a retrospective case-control study, mainly including operation time, postoperative adverse complications, postoperative hospital stay days, and treatment costs. In this way, it provides new ideas and insights for the treatment of gastric submucosal tumors.

1. **Research methodology**
2. Research materials

This study intends to include 120 patients who underwent endoscopic submucosal extraction (ESE) or sleuctomy combined with ESE for the treatment of gastric submucosal masses. By reviewing patient pathology and surgical image records, the baseline characteristics (including age, gender, tumor size, tumor location, and pathological diagnosis), surgical treatment process (including tumor exposure time, tumor resection time, and EFTR conversion rate), adverse events (postoperative perforation, bleeding, fever, abdominal pain), and treatment costs were retrospectively collected.

1. Research content

R software was used to perform statistical analysis of relevant information. The calculation data were expressed as "examples (percentages)", and the differences between the two groups were compared by chi-square test or Fisher's exact test. The ecumenical data is expressed as "mean ± (standard error)", and if the normal distribution is met, the T test is used, otherwise the rank sum test is used to compare the differences between the two groups. P<0.05 indicates a statistical difference.

1. **Findings**

A retrospective case-control research paper was written to illustrate whether endoscopic mucosal snare resection combined with ESE has higher efficacy and safety in the treatment of gastric submucosal tumors.
